# C-reactive protein (CRP) and Erythrocyte Sedimentation Rate (ESR) Trends following Total Hip and Knee Arthroplasties in an Indian Population – A Prospective Study

**DOI:** 10.5704/MOJ.2107.021

**Published:** 2021-07

**Authors:** A Krishna, S Garg, S Gupta, H Bansal

**Affiliations:** 1 Department of Orthopaedics, All India Institute of Medical Sciences, New Delhi, India; 2 Department of Orthopaedics, Government Medical College and Hospital, Chandigarh, India; 3 Department of Orthopaedics, Fortis Hospital Mohali, Chandigarh, India

**Keywords:** ESR, CRP, total hip arthroplasty, total knee arthroplasty, Indian population

## Abstract

**Introduction:**

To evaluate the trends of C-reactive protein (CRP) and Erythrocyte sedimentation rate (ESR) in the first three weeks after uncomplicated total hip (THR) and total knee (TKR) arthroplasty/replacement in the Indian population and to compare it with available literature.

**Materials and methods:**

A total of 90 patients were enrolled for this prospective study, of which 30 were unilateral THR, 30 were unilateral TKR (U/L TKR) and 30 were simultaneous bilateral TKR (B/L TKR). Serum CRP and ESR were measured on the day before surgery and post-operatively on day 1st, 2nd, 3rd, 7th, 12th, and at the end of 3rd week.

**Results:**

CRP showed a peak at day 2nd with normalisation to pre-operative value by the end of 3rd week. While ESR showed a peak on day 3rd and continued to remain elevated even at end of 3rd week post-operatively. Both mean CRP and ESR values were higher in THR patients followed by in B/L TKR and then in U/L TKR patients.

**Conclusion:**

CRP persists to be the best acute phase reactant in the early post-operative phase with a relatively typical pattern as compared to ESR. CRP values peak at post-operative day 2nd and then show a gradual decline. However, its normalisation to pre-operative baseline values may vary among different groups of population.

## Introduction

Periprosthetic infection following total joint arthroplasty is a major complication. Although it occurs in a small percentage of patients (<1%), it results in substantial morbidity and compromised functional outcomes^1^. The diagnosis of periprosthetic infection requires a high index of clinical suspicion, serological investigations, diagnostic imaging, and microbiological analysis^1,2^.

In this setting serological inflammatory makers specifically, erythrocyte sedimentation rate (ESR) and C-reactive protein (CRP) are often used as an initial preliminary diagnostic as well as follow-up tools to monitor the response to therapy, since they are inexpensive, non-invasive, and widely available tests^3-6^.

The role of CRP and ESR as acute phase reactants in the early perioperative phase is well studied and established^3-8^. Normal temporal values of these biomarkers in the early perioperative phase following total hip and knee arthroplasty have been studied widely in the literature^9-13^. While ESR continues to remain elevated for a longer duration in the post-operative period following a replacement surgery, CRP does follow a regular pattern with an immediate peak at 2nd post-operative day followed by a gradual decline over a period of time^9,12,13^. However, the time taken for the normalisation of CRP values to its pre-operative level may vary and differs among various studies involving different populations^14,15^. It is important to realise that a single CRP or ESR reading holds very limited value and that a trend must be observed to maximise its full usefulness. The present study was conducted to evaluate this normal temporal distribution of these two most commonly used acute phase reactants in the early perioperative phase following uncomplicated total hip and knee replacement surgeries in an Indian population and to compare the trend with the available literature.

## Materials and Methods

This was a prospective study, conducted at a tertiary level teaching institute after approval from the Institute Ethical Committee. A written, informed consent were obtained from all the patients enrolled in the study.

The aims of the present study was to evaluate the normal trend and natural kinetics of CRP and ESR in acute phase (<3 weeks) after uncomplicated total hip and total knee replacement surgeries in an Indian population and to compare the trend with the available literature.

A total of 90 patients (30 unilateral THR, 30 unilateral TKR, and 30 simultaneous bilateral TKR) were enrolled in this study. Patients having uncomplicated osteoarthritis of hip or knee requiring total hip or knee replacement were included in the study. Only ASA Grade 1 patients were enrolled to avoid confounding factors such as co-morbidities affecting the values of acute-phase reactants during the perioperative phase. Patients with osteoarthritis secondary to established rheumatoid arthritis or patients showing pre-operative clinical features of any systemic inflammatory arthropathies were excluded from the study. Patients having abnormal preoperative values of serum CRP or ESR or both were also excluded from the study.

All total hip surgeries were performed via a standard posterior approach and all total knee replacement surgeries were performed via a medial parapatellar approach through a straight midline incision. Blood samples were obtained on a day before operation and at 1st, 2nd, 3rd, and 7th post-operative day. Further samples were also drawn at the time of suture removal on 12th day, and at the time of first follow-up at the end of 3rd week.

A total of 2ml of a blood sample for each CRP and ESR analysis was collected. Quantitative CRP analysis was done by employing a testing kit which was based on the principle of immunoassay with a normal reference range of 0 to 5mg/l. ESR was done using the Wintrobes method with a normal reference range of 0 to 30mm/hr. Prophylactic antibiotic therapy was instituted on the morning of the day of surgery and continued for three days after the surgery as per the Institute protocol.

Discrete categorical data was represented in the form of either a number or a percentage (%). Continuous data was written in the form of its mean and standard deviation. The normality of quantitative data was checked by measures of Kolmogorov–Smirnov tests of normality. For normally distributed data means of three groups of outcomes were compared using One–Way ANOVA followed by the Post Hoc Multiple Comparison test. For skewed data Kruskal-Wallis test followed by the Mann–Whitney test for two groups was applied. Wilcoxon signed-rank test was applied to compare time-related scores. The reference range for uneventful cases was decided based on a 95% confidence interval, performed at a significance level of 0.05. All analyses were performed using Statistical Package for the Social Sciences [SPSS version 17].

## Results

The mean age of patients undergoing THR was 47.32 years, whereas in bilateral TKR and unilateral TKR the mean age was 61.8 years and 64.1 years, respectively. The unilateral TKR group had an equal distribution of male and female population groups (50% each), the bilateral TKR group had 84.6% female population and 15.4% male population, whereas the THR group had 71% male population and 29% female population.

**Fig. 1:**
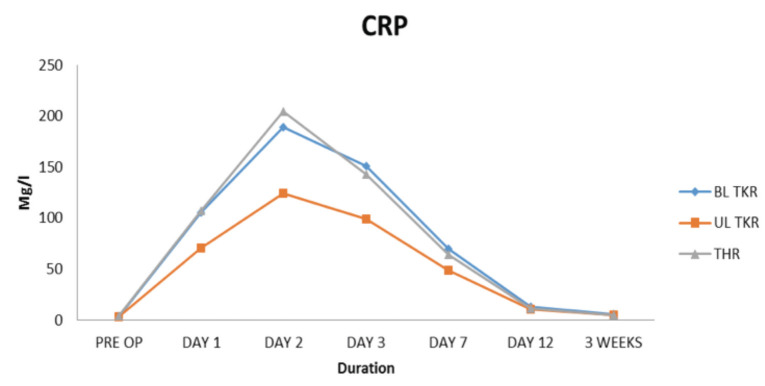
Graph depicting the CRP curve in three groups

**Fig. 2:**
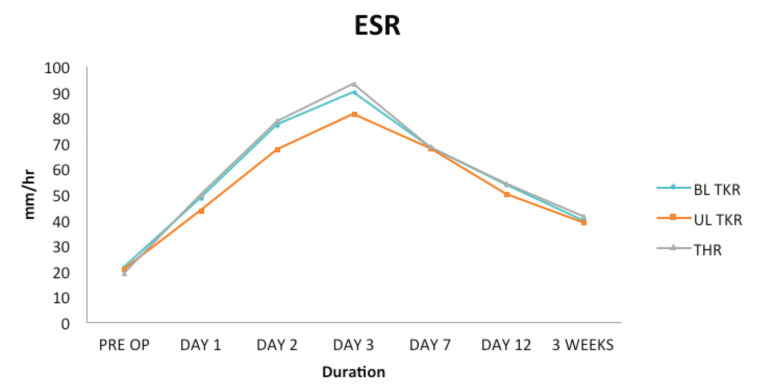
Graph depicting the ESR curve in three groups.

In the THR group, the CRP curve showed a peak at day 2nd with a mean value of 204.88mg/l followed by a decreasing trend after day 3rd. Though the decreasing trend of CRP started after day 3rd, the fall in CRP level between day 3rd and day 7th was greater than between day 7th and day 12th (Table I and Fig. 1). ESR reached a peak on day 3rd with a mean value of 93.25mm/hr. Though a decreasing trend in ESR values was observed after day 3rd, the value of ESR remained elevated at end of 3rd week post-operatively with a mean of 41.48mm/hr (Table II and Fig. 2).

**Table I:** Showing That Peak CRP values in all three groups is reached at day 2. The mean peak values are highest in THR group and lowest in unilateral TKR group. Normalisation of values is seen at the end of three weeks

Mean ± SD CRP (Mg/l)							
	Pre-Op ± SD	Day 1 ± SD	Day 2 ± SD	Day 3 ± SD	Day 7 ± SD	Day 12 ± SD	3 Weeks ± SD
BL TKR	3.49±1.08	105.30±22.99	189.56±33.27	150.76±57.25	70.15±14.15	13.08±5.52	5.22±2.25
UL TKR	3.19±1.55	70.76±23.63	124.36±35.50	99.03±36.08	48.76±12.96	10.33±2.57	4.79±2.42
THR	4.10±1.46	107.37±28.51	204.88±45.69	142.85±67.30	64.14±13.33	11.62±2.61	5.12±2.05

**Table II:** Showing that peak value of ESR is reached at day 3 in all three groups. Mean peak value is highest in THR group and lowest in unilateral TKR group. Values of ESR continue to remain elevated at three weeks

Mean ±SD ESR (mm/hr)							
	Pre-Op ± SD	Day 1 ± SD	Day 2 ± SD	Day 3 ± SD	Day 7 ± SD	Day 12 ± SD	3 Weeks ± SD
BL TKR	21.53±4.16	48.28±5.05	76.95±5.31	89.73±6.15	68.36±6.28	53.56±5.20	39.87±4.57
UL TKR	20.56±5.07	43.37±4.44	67.56±6.07	81.31±4.82	68.00±4.61	50.00±6.31	38.81±3.98
THR	19.00±3.54	49.51±5.87	78.71±6.41	93.25±6.40	68.61±4.83	54.07±5.33	41.48±4.03

In the bilateral TKR group (performed simultaneously as a one-stage procedure) the trend of serum CRP levels was similar to that of the THR group. The mean values of CRP in B/L TKR group were slightly lower than the THR group with no statistically significant difference between the two groups (Table I and Fig. 1). ESR also followed the same trend in the bilateral TKR group like that in the THR group with a mean peak value on day 3rd which was only marginally lower than that of the THR group (Table II and Fig. 2). ESR remained elevated at end of 3rd week post-operatively with a mean of 39.87mm/hr.

On evaluating the unilateral TKR group, though the trend of serum CRP and ESR was similar to that of bilateral TKR and THR group, mean values of both CRP and ESR were much lower and statistically significant (p value<.05) as compared to that of bilateral TKR or THR group (Table I and Table II). Also, similar to the bilateral TKR and THR groups, a downward trend of serum CRP started after day 3rd (Table I and Fig. 1). Serum CRP levels normalised at the end of 3rd week with a mean of 4.79mg/l. ESR, however, reached a peak on day 3rd with a mean of 81.31mm/hr and remained elevated at the end of 3rd week post-operatively with a mean of 38.81mm/hr (Table II and Fig. 2).

When multiple comparisons were made between the THR group and bilateral TKR group keeping the dependent variable as serum CRP or ESR, no statistically significant difference (p-value >0.05) was noted between these two groups on day 1st, 2nd, 3rd, 7th, 12th, or at the end of 3rd week, suggesting that the mean values of serum CRP and ESR levels follow a similar pattern in both these groups (Table III and Table IV).

**Table III:** Multiple comparison between the three groups keeping the dependent variable CRP

TIME	(I) GROUP	(J) GROUP	Mean (I-J)	Std. Error Difference	Significance (P value)	95% Confidence Interval	
						Lower Bound	Upper Bound
Pre-Op	BL TKR	UL TKR	.3024	.4321	1.000	-.757	1.362
		THR	-.6071	.3616	.293	-1.494	.280
	UL TKR	BL TKR	-.3024	.4321	1.000	-1.362	.757
		THR	-.9095	.4186	.100	-1.936	.117
	THR	BL TKR	.6071	.3616	.293	-.280	1.494
		UL TKR	.9095	.4186	.100	-.117	1.936
Day 1	BL TKR	UL TKR	34.5313(*)	8.1435	.000	14.556	54.506
		THR	-2.0710	6.8156	1.000	-18.789	14.647
	UL TKR	BL TKR	-34.5313(*)	8.1435	.000	-54.506	-14.556
	THR	THR BL TKR	-36.6022(*) 2.0710	7.8894 6.8156	.000 1.000	-55.954 -14.647	-17.251 18.789
Day 2	BL TKR	UL TKR UL TKR	36.6022(*) 65.2067(*)	7.8894 12.5512	.000 .000	17.251 34.420	55.954 95.993
		THR	-15.3172	10.5045	.448	-41.083	10.449
	UL TKR	BL TKR	-65.2067(*)	12.5512	.000	-95.993	-34.420
		THR	-80.5240(*)	12.1595	.000	-110.350	-50.698
	THR	BL TKR	15.3172	10.5045	.448	-10.449	41.083
		UL TKR	80.5240(*)	12.1595	.000	50.698	110.350
Day 3	BL TKR	UL TKR	51.7341(*)	18.5043	.020	6.346	97.123
		THR	7.9138	15.4869	1.000	-30.074	45.901
	UL TKR	BL TKR	-51.7341(*)	18.5043	.020	-97.123	-6.346
		THR	-43.8204	17.9268	.050	-87.792	.152
	THR	BL TKR	-7.9138	15.4869	1.000	-45.901	30.074
		UL TKR	43.8204	17.9268	.050	-.152	87.792
Day 7	BL TKR	UL TKR	21.3925(*)	4.3076	.000	10.826	31.959
		THR	6.0066	3.6052	.300	-2.836	14.850
	UL TKR	BL TKR	-21.3925(*)	4.3076	.000	-31.959	-10.826
		THR	-15.3859(*)	4.1732	.001	-25.622	-5.150
	THR	BL TKR	-6.0066	3.6052	.300	-14.850	2.836
		UL TKR	15.3859(*)	4.1732	.001	5.150	25.622
Day 12	BL TKR	UL TKR	2.7510	1.2420	.090	-.295	5.797
		THR	1.4594	1.0395	.494	-1.090	4.009
	UL TKR	BL TKR	-2.7510	1.2420	.090	-5.797	.295
		THR	-1.2915	1.2032	.860	-4.243	1.660
	THR	BL TKR	-1.4594	1.0395	.494	-4.009	1.090
		UL TKR	1.2915	1.2032	.860	-1.660	4.243
At 3	BL TKR	UL TKR	.4293	.7018	1.000	-1.292	2.151
Weeks		THR	.0973	.5874	1.000	-1.343	1.538
	UL TKR	BL TKR	-.4293	.7018	1.000	-2.151	1.292
		THR	-.3321	.6799	1.000	-2.000	1.336
	THR	BL TKR	-.0973	.5874	1.000	-1.538	1.343
		UL TKR	.3321	.6799	1.000	-1.336	2.000

* The mean difference is significant at the .05 level.

**Table IV:** Multiple comparison between the three groups keeping the dependent variable ESR

Time	(I) Group	(J) Group	Mean Difference (I-J)	Std. Error	Significance (p value)	95% Confidence Interval	
						Lower Bound	Upper Bound
Pre-Op	BL TKR	UL TKR	.9760	1.3147	1.000	-2.249	4.201
		THR	2.5385	1.1003	.072	-.160	5.237
	UL TKR	BL TKR	-.9760	1.3147	1.000	-4.201	2.249
		THR	1.5625	1.2737	.672	-1.562	4.687
	THR	BL TKR	-2.5385	1.1003	.072	-5.237	.160
		UL TKR	-1.5625	1.2737	.672	-4.687	1.562
Day 1	BL TKR	UL TKR	4.9096(*)	1.6855	.014	.775	9.044
		THR	-1.2283	1.4106	1.000	-4.688	2.232
	UL TKR	BL TKR	-4.9096(*)	1.6855	.014	-9.044	-.775
		THR	-6.1379(*)	1.6329	.001	-10.143	-2.133
	THR	BL TKR	1.2283	1.4106	1.000	-2.232	4.688
		UL TKR	6.1379(*)	1.6329	.001	2.133	10.143
Day 2	BL TKR	UL TKR	9.3952(*)	1.8964	.000	4.744	14.047
		THR	-1.7584	1.5872	.815	-5.652	2.135
	UL TKR	BL TKR	-9.3952(*)	1.8964	.000	-14.047	-4.744
		THR	-11.1536(*)	1.8372	.000	-15.660	-6.647
	THR	BL TKR	1.7584	1.5872	.815	-2.135	5.652
		UL TKR	11.1536(*)	1.8372	.000	6.647	15.660
Day 3	BL TKR	UL TKR	8.4183(*)	1.9085	.000	3.737	13.100
		THR	-3.5208	1.5973	.092	-7.439	.397
	UL TKR	BL TKR	-8.4183(*)	1.9085	.000	-13.100	-3.737
		THR	-11.9391(*)	1.8490	.000	-16.474	-7.404
	THR	BL TKR	3.5208	1.5973	.092	-.397	7.439
		UL TKR	11.9391(*)	1.8490	.000	7.404	16.474
Day 7	BL TKR	UL TKR	.3615	1.7027	1.000	-3.815	4.538
		THR	-.2578	1.4251	1.000	-3.753	3.238
	UL TKR	BL TKR	-.3615	1.7027	1.000	-4.538	3.815
		THR	-.6194	1.6496	1.000	-4.666	3.427
	THR	BL TKR	.2578	1.4251	1.000	-3.238	3.753
		UL TKR	.6194	1.6496	1.000	-3.427	4.666
Day 12	BL TKR	UL TKR	3.5615	1.7517	.137	-.735	7.858
		THR	-.5159	1.4661	1.000	-4.112	3.080
	UL TKR	BL TKR	-3.5615	1.7517	.137	-7.858	.735
		THR	-4.0774	1.6970	.057	-8.240	.085
	THR	BL TKR	.5159	1.4661	1.000	-3.080	4.112
		UL TKR	4.0774	1.6970	.057	-.085	8.240
At 3 Weeks	BL TKR	UL TKR	1.0606	1.3427	1.000	-2.233	4.354
		THR	-1.6108	1.1237	.469	-4.367	1.146
	UL TKR	BL TKR	-1.0606	1.3427	1.000	-4.354	2.233
		THR	-2.6714	1.3008	.131	-5.862	.519
	THR	BL TKR	1.6108	1.1237	.469	-1.146	4.367
		UL TKR	2.6714	1.3008	.131	-.519	5.862

* The mean difference is significant at the .05 level.

When multiple comparisons were made between the THR group and the unilateral TKR group, keeping the dependent variable as serum CRP, statistically significant differences (p-value <0.05) were noted between these two groups on day 1st, 2nd, 3rd, and 7th. However, no statistically significant difference (p-value >0.05) was seen at day 12th or at the end of 3rd week. A similar observation was made between the bilateral TKR and the unilateral TKR group (Table III).

When the dependent variable was kept as ESR, statistically significant differences (p-value <0.05) between THR and unilateral TKR group were noted only on day 1st, 2nd, and 3rd. No statistically significant difference was noted on day 7th, day 12th, and at the end of 3rd week. A similar observation was made between the bilateral TKR and the unilateral TKR group (Table IV).

## Discussion

Infection is a persistent recurring fear for the orthopaedic surgeon performing total joint replacements. Nonspecific clinical symptoms and signs make it difficult to suspect and diagnose infection in the early perioperative period. Thus, we frequently need objective tests to exclude the possibility of infection.

C-reactive protein and ESR have been widely used to determine the presence of infection after total joint replacement, but appropriate quantitative interpretation of the values and trend of CRP and ESR may be difficult during the early perioperative phase. This is because CRP and ESR being acute phase reactants also show a normal physiological response curve in the immediate post-operative period due to surgery-induced tissue damage, which can cause their elevation in the acute phase following surgery.

In the present study, CRP and ESR differ in normal temporal patterns of post-operative levels after uncomplicated THR and TKR procedures. The temporal changes of CRP values were faster and greater than those of ESR. The number of fold changes in the CRP levels was much greater than the number of fold changes in the ESR levels. CRP level rapidly reached a peak on day 2nd and thereafter the levels decreased in a biphasic pattern. The first phase occurred after day 3rd when CRP levels decreased rapidly, and the second phase came after day 7th with a gradual decrease until normalisation at the end of 3rd week. In contrast, ESR levels peaked on 3rd day and gradually decreased but remained elevated above the normal reference level (0-30mm/ hr) at the end of 3rd week. These findings are in concordance with previous studies in the literature evaluating the postoperative levels of CRP and ESR after TKR or THR^12,13^. However, in the present study, CRP trend in THR and TKR in the Indian population differed from those published in the literature in some aspects.

In studies by White *et al*^10^ and Bilgen *et al*^12^, the CRP trend of total hip and total knee replacement shows mean values of CRP to be higher in TKR than in the THR surgeries. In another study by Niskanen *et al*^11^, the CRP trend after total hip and knee replacement, followed the same trend as the above two studies with mean values of CRP in the TKR group higher than the THR group. But, the present study shows mean values of CRP to be higher and statistically significant in the THR group as compared to the U/L TKR group and comparable to the B/L TKR group. This difference could be attributed to the relatively younger patient population in the THR group as compared to the above-mentioned studies. In the Asian population, patients undergoing THR are relatively younger as the incidence of secondary hip osteoarthritis necessitating total hip replacement is higher as compared to the western population^16^.

The mean peak value of CRP at day 2nd following THR and TKR was found to be in concordance with that in the previous literature^9-12^. However, the normalisation pattern of CRP to its baseline or pre-operative values varied across the different studies with different population characteristics.

The normalisation of CRP after a replacement surgery is of paramount importance for the operating surgeon as it is the rising trend of CRP after touching the normal baseline values that help alert the surgeon for the possibility of delayed infection. It, therefore, becomes imperative to know the period in which CRP touches the normal baseline values after replacement surgery.

In the present study, CRP values were normalised to preoperative values by the end of 3rd week following THR and TKR. The normalisation period of CRP for THR in the present study was in concurrence with the study done by Aalto *et al*^9^ and Bilgen *et al*^12^. Although, in the study by Aalto *et al*^9^, the decreasing trend of CRP after THR was noted only after day 7th as compared to day 3rd in the present study, CRP values were invariably normalised at the end of 3rd week in both the studies.

When normalisation period of CRP in TKR was evaluated it was observed that it touched the pre-operative baseline value only at the end of 8 weeks in the study by Bilgen *et al*^12^ as compared to the end of 3rd week in the present study. In a study by Park *et al*^13^ temporal values of CRP after TKR reached the normal reference at the end of 6th week. In another study by Londhe *et al*^14^, CRP levels after unilateral TKR reached the normal baseline level after 12 weeks and at the end of 16 weeks after bilateral TKR. In an analysis done on the Iranian population by Nazem *et al*^15^, CRP levels after TKR and THR did not reach the normal baseline values even after one year of the procedure.

We believe that this variation in the normalisation of CRP values could be attributed to a multitude of factors. Besides the well- known factors such as the surgical approach used in replacement surgery, the degree of surgical dissection performed, the duration of surgery, and post-operative rehabilitation protocol^17^, the demographical and geographical characteristics of the population may also seem to play an important contributing role in the CRP and ESR trends after an arthroplasty procedure. The operating surgeon should be wary of these factors and CRP and ESR trend after an arthroplasty procedure should be interpreted in the light of the above elements before making any decision.

Though there are numerous studies in the literature evaluating CRP and ESR trends post total hip and knee replacement surgeries, very few have actually evaluated the effect of the racial factors affecting CRP values^18^. Most of the studies have primarily been done on a subset of the european population^9-13^ and to the best of the author's knowledge, very few have studied the early post-operative trend of CRP and ESR on a subset of Asian population^14,15^. The present study is an attempt to fill this gap in literature by evaluating the CRP and ESR trend in an Indian population. Also, most of the published literature has evaluated CRP and ESR trends in THR and unilateral TKR, whereas the present study has gone a step further by evaluating the CRP and ESR trends in THR, unilateral TKR, and simultaneous bilateral TKR.

This prospective study also confirms and highlights the facts previously recorded in the literature^19-25^ that CRP correlates with a higher degree of inflammatory activity with a more rapid increase and a faster return to normal than ESR at the end of 3rd week. CRP shows a more predictable response with less atypical patterns and appears to be a better indicator of acute-phase response than ESR.

CRP and ESR levels in THR and B/L TKR follow a similar trend with comparable values suggesting that surgical trauma induced by these two surgeries appears to be the same. However, CRP and ESR levels after unilateral TKR, though following a similar trend as THR and simultaneous bilateral TKR groups, had much lower mean values, suggesting that the surgical trauma induced in U/L TKR is much less than the other two groups.

There are however, a few limitations of the present study. This was a single institute prospective study done on a subset of Indian population. The cohort of patients selected may not be representative of the geographical and demographical characteristics of the pan Indian population. In the present study, ESR was not followed longer than three weeks to see when it was normalised. Also, since ESR was not followed long enough it could not be compared with other studies in the literature which had a longer follow-up period of ESR.

## Conclusion

CRP persists to be the best acute phase reactant in the early post-operative phase with a relatively typical pattern as compared to ESR. CRP values peak at post-operative day 2nd and then show a gradual decline. However, its normalisation to pre-operative values may vary among different groups of population.

In the light of this fact, authors suggest that large multicentric studies involving different population groups may validate the plausible effect of demographical and geographical factors on CRP and ESR trends after total hip and knee arthroplasty.
